# Supplementary material for: Inactivated rabies virus vectored SARS-CoV-2 vaccine prevents disease in a Syrian hamster model
Source: PLoS Pathog. 2021 Mar 25;17(3):e1009383. doi: 10.1371/journal.ppat.1009383 (PMC8023494; doi:10.1371/journal.ppat.1009383)
Supplement: S1 Table — (DOCX) [file ppat.1009383.s001.docx]

**Supplemental Table 1.** Criteria for histopathology scoring

|  | **Scores🡪** | **0** | **1** | **2** | **3** | **4** |
| --- | --- | --- | --- | --- | --- | --- |
| **A** | Extent of inflammation (% tissue involved) | 0 | <10 | 10-30 | 30-60 | >60 |
| **B** | Inflammatory foci type | No inflammation | Patchy inflammatory foci, few (<2) | Patchy inflammatory foci, many (>2) | Large inflammatory foci, few (<2) | Large inflammatory foci, many (>2) |
| **C** | Alveolar septa | Thin and delicate | Thickened in <10% HPF | Thickened in <30% HPF | Thickened in <60% HPF | Thickened in >60% HPF |
| **D** | Airways | Clear; no cells | Few cells in airway | Moderate cells in airway | More cells in air way; epithelial hyperplasia | Occlusion of air way/epithelial hyperplasia or desquamation |
| **E** | Alveoli/ perivascular cuff/blood vessels/ pleuritis/cell types | Clear; no inflammatory cells | Few cells; few PMN or MNC | Moderate cells/ PVC/mild congestion/  mild pleuritis/mostly MNC | More cells/PVC/ more congestion and pleuritis/more MNC and PMN | Abundant cells/large PVC/severe congestion or pleuritis/mixed cells |

Adapted from Matute-Bello et al., 2011 [1].

HPF – high power field (>10x); PMN – polymorphonuclear cells/heterophils; MNC – mononuclear cells including lymphocytes and macrophages; PVC – perivascular cuff.

1. Matute-Bello G, Downey G, Moore BB, Groshong SD, Matthay MA, Slutsky AS, et al. An official American Thoracic Society workshop report: features and measurements of experimental acute lung injury in animals. Am J Respir Cell Mol Biol. 2011;44(5):725-38. Epub 2011/05/03. doi: 10.1165/rcmb.2009-0210ST. PubMed PMID: 21531958; PubMed Central PMCID: PMCPMC7328339.
